# The stuff that motor chunks are made of: Spatial instead of motor representations?

**DOI:** 10.1007/s00221-015-4457-8

**Published:** 2015-10-20

**Authors:** Willem B. Verwey, Eduard C. Groen, David L. Wright

**Affiliations:** MIRA Research Institute, University of Twente, Enschede, The Netherlands; Human Performance Laboratories, Department of Health and Kinesiology, Texas A&M University, College Station, TX USA; Fraunhofer Institute for Experimental Software Engineering, Kaiserslautern, Germany; Department of Cognitive Psychology and Ergonomics, University of Twente, PO Box 217, 7500 AE Enschede, The Netherlands

**Keywords:** Cognitive models, Motor control, Sequence learning, Motor chunking, Spatial reference frames

## Abstract

In order to determine how participants represent practiced, discrete keying sequences in the discrete sequence production task, we had 24 participants practice two six-key sequences on the basis of two pre-learned six-digit numbers. These sequences were carried out by fingers of the left (L) and right (R) hand with between-hand transitions always occurring between the second and third, and the fifth and sixth responses. This yielded the so-called LLRRRL and RRLLLR sequences. Early and late in practice, the keypad used for the right hand was briefly relocated from the front of the participants to 90° at their right side. The results indicate that after 600 practice trials, executing a keying sequence relies heavily on a spatial cross-hand representation in a trunk- or head-based reference frame that after about only 15 trials is fully adjusted to the changed hand location. The hand location effect was not found with the last sequence element. This is attributed to the application of explicit knowledge. The between-hand transitions appeared to induce initial segmentation in some of the participants, but this did not consolidate into a concatenation point of successive motor chunks.

## Introduction

A large number of studies has been devoted to understanding how humans automate movement sequences like writing one’s signature and shifting gears when driving an automobile (for recent reviews, see, e.g., Abrahamse et al. 2010, 2013; Heuer and Massen 2013; Keele et al. 2003; Shea and Kovacs 2013; Verwey et al. 2015). Automating movement sequences allow individuals to devote most of their processing resources to concurrent tasks and/or prepare for upcoming behavior while they are executing a familiar movement pattern. To study the human capacity to automate movement sequences, researchers have developed various laboratory tasks that allow them to distinguish the various cognitive processes and representations underlying skilled movement execution. These tasks include, among others, sequential aiming tasks like the flexion–extension (FE) task (Panzer et al. 2006) and sequential key pressing tasks like the serial reaction time (RT) task (Nissen and Bullemer 1987) and the presently used *discrete sequence production* (DSP) task (Abrahamse et al. 2013; Verwey 1999). Research with tasks like these have made a strong case that sequential motor learning involves the simultaneous development and use of various task- and practice-dependent representations (Berniker et al. 2014; Keele et al. 1995; Panzer et al. 2014; Proteau and Carnahan 2001; Shea and Kovacs 2013; Shea et al. 2011; Verwey et al. 2015; Wiestler et al. 2014). Representing movement sequences in such a redundant way allows human motor control to be flexibly adjusted to changes in the environment and the effectors being used, as well as deal with the detrimental effects on serial behavior of aging and neural damage.

In the laboratory, movement order in sequence learning tasks is usually guided by a series of stimuli, each indicating an element of the sequence. Consequently, sequences in these tasks are initially produced in the *reaction mode* (Abrahamse et al. 2013; Hikosaka et al. 1999; Park and Shea 2005; Verwey 2003). As the movement sequence is repeated, regularities in the order of the movements—and the associated stimuli—are gradually learned at all levels of information processing (Abrahamse et al. 2010; Verwey et al. 2015). With limited practice, participants still need external guidance to produce the sequence, but the successive processes and movements are already primed using the *associative mode* (Verwey and Abrahamse 2012; also see Ruitenberg et al. 2014; Verwey and Wright 2014). The associative mode is assumed to be responsible for the skill that develops in the serial RT task.

When movement sequences are limited to about 5–8 elements, as is typically the case for the DSP task (Abrahamse et al. 2013), they can be planned before being executed. This allows strategies to develop and use effective representations (Henry and Rogers 1960; Sternberg et al. 1978; Verwey 1996). Consequently, with further practice, the need for perceptual and central processing resources to translate individual stimuli (S) into responses (Rs) reduces because sequence-specific representations develop that are called *motor chunks*, and that rely on R–R associations. These motor chunks allow sequence execution to occur without external guidance in the so-called *chunking mode* (Abrahamse et al. 2013; Verwey and Abrahamse 2012). Like memory chunks in general (Halford et al. 1998; Miller 1956; Newell and Rosenbloom 1981), motor chunks help bypass limitations in information processing so that short movement series can be selected, prepared, and executed as if they constitute a single response (Verwey 1999). While the motor chunk construct is generally accepted, it is defined by its behavioral properties, and despite its name, it remains unclear as to how motor chunks code the individual movements of the DSP task (Verwey 2015). This is the central issue in the present paper.

### Sequence representations

The recently proposed cognitive framework for sequential motor behavior (C-SMB; Verwey et al. 2015) postulates that the repeated execution of movement sequences induces the development of a number of different representations some of which may underlie motor chunks. In keeping with the general notion that with practice perceptual motor skills become increasingly task-specific (Ackerman 1988; Fleishman and Hempel 1955; Newell and Rosenbloom 1981; Proteau et al. 1992), C-SMB assumes that, first, S-R associations develop that allow a central processor to perform in the reaction mode. With limited practice, *central*-*symbolic* (e.g., spatial and/or verbal) representations develop that can be parsed by the central processor in a cognitive loop. Central-symbolic representations may involve various ways of coding the sequence, such as a visual–spatial image of the required actions (relying on episodic memory), the numbers of one’s PIN (using verbal memory), and/or a spatial representation of successive target locations (spatial memory). This type of knowledge can probably be verbally expressed and is assumed to contribute heavily to what has been called explicit sequence knowledge. Given the abstract level of this knowledge, it is independent of the effector system being used.

Spatial representations of the successive movements are assumed to develop more slowly than the verbal representations that are stored in episodic memory (Shea et al. 2011; Witt et al. 2008). However, during sequence execution, these spatial representations require less cognitive processing than verbal descriptions because spatial representations provide information that can directly be used for planning each individual movement. Inspired by findings with single-cell recordings, behavioral research has explored the nature of these spatial representations in various tasks. That research confirmed neurophysiological indications for a variety of spatial reference frames in which movements can be coded (Flanders and Soechting 1995; Shea et al. 2011; Wiestler et al. 2014). That is, the spatial coordinates of individual movement endpoints may be relative to objects in the person’s environment (they are then coded in an *allocentric reference frame*), but also relative to body parts like the eye, head, shoulder, trunk, forearm, and hand (i.e., involving *egocentric reference frames*, Bernier and Grafton 2010; Colby and Goldberg 1999; Gentilucci et al. 1996; Krakauer et al. 1999; Obhi and Goodale 2005; Shea et al. 2011; Zacks 2008).

Allocentric representations have been argued to support strategic, goal-oriented processes that are effector independent. Egocentric reference frames are important for executing motor sequences (Willingham 1998), may include effector-dependent and effector-independent components (Wiestler et al. 2014), and are associated with other brain networks than allocentric representations (Zacks 2008). It is generally believed now that sequential motor tasks involve the simultaneous use of spatial representations with varying reference frames (Keulen et al. 2002; Leoné et al. 2015; McIntyre et al. 1998). Activating a particular movement goal and action plan would automatically activate the spatial representations needed for executing a sequencing task (Jeannerod 1997). The contributions of the various reference frames can probably also be modulated intentionally (Abrams and Landgraf 1990; Proctor et al. 2004), which might be useful when the movement sequence is carried out in different spatial contexts. Neurophysiological measurements indicate that various brain regions can host different reference frames simultaneously. Also, one reference frame can be replaced by another within less than about 100 ms (Derdikman and Moser 2010; Zacks 2008), which may be associated with the ability to rotate spatial representations (Georgopoulos et al. 1986; Leoné et al. 2015; Pellizzer et al. 2009; Petit et al. 2003; Shepard and Metzler 1971).

According to C-SMB, extensive practice of a sequential motor task would introduce the use of *motor* representations that are carried out by the motor processor. This type of representation is called motoric because it would entail activation patterns of agonist/antagonist muscles (Shea et al. 2011), musculoskeletal forces and dynamics (Krakauer et al. 1999), joint angles (Criscimagna-Hemminger et al. 2003), posture-related representations (Rosenbaum et al. 2009; Rosenbaum et al. 1999), and/or the orientation of body segments relative to each other (Lange et al. 2004). Despite the varying terminology, these notions all suggest that a sequence representation is developed in terms of muscles or muscle groups (cf. Keele 1968). Due to their motoric coding, these representations require little processing to execute a sequence and allow rapid sequence execution by the motor processor without relying very much on the central processor (Verwey et al. 2015). These motor representations are not accessible to processes involved in awareness, that is, motor representations are implicit (though people may still have independent explicit knowledge too, Jeannerod 1997).

There now seems some consensus that the various representations underlying motor sequencing skill develop at different processing levels and that each representation can contribute to the execution of individual movements. Even central-symbolic codes—like verbal ones—and reacting to guiding stimuli are believed to contribute to the execution of highly practiced motor sequences (Keisler and Shadmehr 2010; Kovacs et al. 2009; Stanley and Krakauer 2013; Verwey 1999). In DSP sequences, these central-symbolic representations may speed up especially those sequence elements that are executed more slowly (Verwey 2015). Furthermore, due to primacy and recency effects in memory (Bonanni et al. 2007; Johnson 1991), the speedup by explicit knowledge can be expected especially for the elements at the sequence start and end. Using Monte Carlo simulations, Verwey (2003) showed that simultaneous activity of independent parallel processing systems—each potentially using another representation—increases execution rate as long as there is an overlap in the distributions of the times it takes each system to trigger a response. A racing processor mechanism appears to be a general feature of information processing as it underlies also models of simple RT, response selection, and the processing of redundant signals (Cho and Proctor 2002; Logan 1988; Logan et al. 2014; Miller and Ulrich 2003; Nicoletti et al. 1984; Proctor and Reeve 1988; Ulrich and Miller 1997). Behavioral support for the idea that executing familiar movement sequences involves a race between (processors using) different representations comes from the finding that individual participants had two or three peaks in their RT distribution when they changed from the reaction to the chunking mode (Verwey 2003). This suggests that for some trials, the fastest of several different representations was not applied, and a less efficient one was used to trigger the appropriate sequence element.

The conclusion in behavioral studies that a motor representation developed is usually based on the slowing that is observed when a highly practiced sequence is executed with another effector (e.g., Andresen and Marsolek 2012; Park and Shea 2005; Verwey and Clegg 2005; Verwey and Wright 2004). Such a motor representation should make execution skill independent of the location in which the effector is being used. However, location-independent sequence execution contrasts with the idea that practice makes perceptuomotor skills increasingly task (and thus location) specific (Ackerman 1988; Fleishman and Hempel 1955; Proteau et al. 1992). In this context, it is interesting that De Kleine and Verwey (2009) found that the speed advantage of the practiced over the unpracticed hand in a DSP task reduced when the practiced hand was located on the other side of the participants’ body. This suggests that the advantage of the practiced over the unpracticed hand cannot always be attributed to a motor (muscle-specific) representation and may instead involve a spatial representation that is both effector specific and sensitive to the location of the hand.

The main goal of the present study was to examine bimanual keying sequences in the DSP task to determine the representations underlying sequencing skill earlier and later in practice. To that end, participants practiced a discrete keying sequence with fingers of both hands while the hands were in the normal adjacent position in front of the body. They then relocated the right hand to the side while the left hand remained in its usual location in front of the trunk (like a 1970s or 1980s musician playing on two keyboards at the same time). According to what will be referred to as the *applicability hypothesis*, participants switch off inappropriate representations when their hand is in a novel location. When in that situation, the most applicable representations are hand-based (i.e., muscle-oriented and/or with a hand-based reference frame), and execution rate will suffer little. An alternative account is called the *adjustment hypothesis*. It assumes that with practice, execution is based on highly task-specific sequence representations that can be fine-tuned for a particular task. So, rather than switching off inappropriate representations, representations are adjusted to accommodate a changed hand location, for example, by rotating a spatial representation of the sequence (Shepard and Metzler 1971).

### Chunk boundaries

Another issue in the present study concerns many indications that movement sequences exceeding about 3–5 elements include multiple motor chunks (Acuna et al. 2014; Bo and Seidler 2009; Kornbrot 1989; Kovacs et al. 2009; Verwey and Eikelboom 2003; Wymbs et al. 2012). The prime indicator that a motor chunk is used consists of a relatively slow response followed by several relatively fast responses (Bo et al. 2009; Bo and Seidler 2009; Kennerley et al. 2004; Ruitenberg et al. 2013; Ruitenberg et al. 2015; for a test of various indicators of motor chunks, see Verwey 2001). The estimate of 3–5 elements per motor chunk in discrete keying sequences is remarkably similar to the chunk size for verbal and visuospatial knowledge of 3–4 in working memory (Anderson 2014; Broadbent 1975; Cowan 2000; Logie and Cowan 2015). One could therefore hypothesize that the initial segmentation^1^ of a longer sequence is determined by working-memory capacity and that practice then consolidates these segments into successive motor chunks. Support for this hypothesis comes from studies showing correlations between individual visuospatial working-memory capacity and motor chunk length (Bo et al. 2009; Bo and Seidler 2009; Seidler et al. 2012). While the number of elements per motor chunk does indeed seem to be a prime determinant of where these transitions occur (Abrahamse et al. 2013), other sequence properties may influence the sequential position of this so-called *concatenation point*. Those properties include regularities in the order of the elements (like 123123, De Kleine and Verwey 2009; Jones 1981; Koch and Hoffmann 2000; Kornbrot 1989; Sakai et al. 2003; Verwey and Eikelboom 2003), the use of random versus blocked practice schedules (Wilde et al. 2005), and the occurrence of a pause at a particular sequential position during practice (Stadler 1993; Verwey 1996; Verwey et al. 2009; Verwey and Dronkert 1996).

The occurrence in the present study of between-hand transitions at two fixed sequential positions allowed us to examine the suggestion by Verwey and Eikelboom (2003) that such transitions might in unfamiliar sequences determine segmentation, which then consolidates with practice into the more robust motor chunks. This idea received some support from the finding with unfamiliar keying sequences that six of the eight observed slow elements in a DSP sequence were associated with a between-hand transition (Verwey et al. 2009). A lasting effect of between-hand transitions in DSP sequences would be consistent with the development of hand-specific sequence representations in the serial RT task (Berner and Hoffmann 2008, 2009) and with indications that fingers of one hand can be prepared by a neural system (a network including the basal ganglia), which differs from the one used for two hands (Adam et al. 2003). The question whether between-hand transitions contribute to dividing a keying sequence into different segments in early practice, that may consolidate with practice into the more robust motor chunks, has not been addressed in earlier DSP studies because those studies involved sequence elements being balanced across fingers of different participants, so that between-hand transitions were distributed across all sequential positions.

### The present experiment

The applicability and the adjustment hypotheses were tested by having participants first practice two six-element DSP sequences with both hands in the typical adjacent frontal location and then examining the effect on the individual sequence elements of relocating the right hand to the side. This was done after approximately 100 and 600 practice trials per sequence. The applicability hypothesis predicts that all sequence elements are slowed when one hand has been relocated because one or more sequence representations are no longer used. This slowing should not change significantly during a test block because new sequence representations do not develop very fast. In contrast, the adjustment hypothesis predicts that the slowing caused by relocating one hand to the side may last for just a few trials because participants quickly learn to apply a transformation to the representations used (like when rotating a spatial representation). This slowing should occur before the first of the responses given by a particular hand because (the representations making up) motor chunks are adjusted before they can be used. Because practice execution is based on fewer, more sequence-specific, representations (Ackerman 1988; Fleishman and Hempel 1955; Newell and Rosenbloom 1981; Proteau et al. 1992), indications for such an adjustment of representations are expected to be stronger in Block 9 than in Block 2. We also assessed awareness of the sequences to determine whether explicit sequence knowledge may contribute to sequence execution. In discrete keying sequence, this may concern especially the second and last responses (i.e., R_2_ and R_6_, Verwey 2015). Finally, we addressed in the conditions in which the right hand was in the front, whether the between-hand transitions might influence the segmentation of each sequence in early practice, and whether this would consolidate with practice when motor chunks develop.

## Method

### Participants

Twenty-four students (16 female, M age = 21 years, SD = 2.0 years) from the University of Twente took part in this study in exchange for course credit. All participants were self-proclaimed right-handed students. Informed consent was obtained from all individual participants included in the study. The study had been approved by the Ethics Committee of the University of Twente and was performed in accordance with the ethical standards described in the Declaration of Helsinki.

### Apparatus

The experiment was programmed and conducted in E-Prime 2.0 on a 2.8 GHz Pentium 4 PC with 512 MB RAM running under Windows XP. Key-specific stimuli consisted of eight squares that subtended a visual angle of approximately 1°. They were presented on a 15″ Philips 107T5 CRT display at a refresh rate of 75 Hz, with a resolution of 640 × 480 pixels, and at 16-bit color depth.

**Fig. 1:**
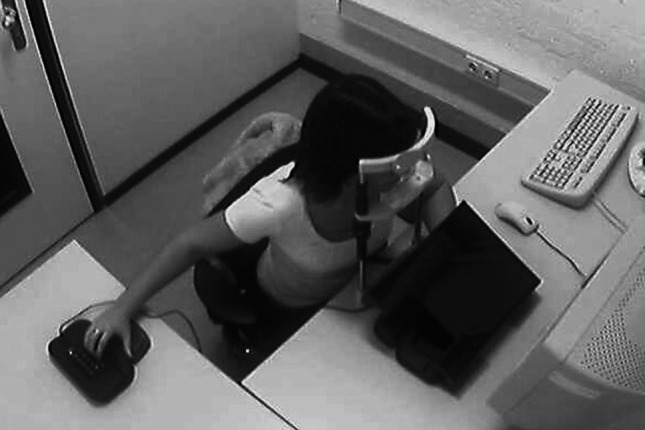
Participant in the test phase condition with the *right hand* on the side, the *left hand* on the keypad that was occluded from vision, and the head in the chinrest

Directly in front of the participant were two Trust USB numeric keypads, embedded near each other in a black wooden mold (see Fig. 1). In this frontal location, the left keypad was itself rotated 90° counterclockwise, the one on the right 90° clockwise. This allowed participants to place their left hand’s little, ring, middle, and index fingers on the left keypad’s “/,” “8,” “5,” and “2” keys, respectively, and their right hand’s index, middle, ring and little fingers on the right keypad’s “3,” “6,” “9,” and “*” keys, respectively. Connecting these two identical keypads via the same USB controller in the computer induced negligible delays (USB 2.0 delays signals by about 8 ms). The dual keypad setup was chosen so that the hands were placed as close together as possible during the practice phase, while in the test phase the right-hand keypad could be relocated without a possible confound with using different keypads. Because participants had their head in a chinrest, the keypad located at the side of the participant was outside the participants’ field of view. To prevent confounding of keypad location and sight at the keypads in the frontal location, we occluded the participants’ sight for the frontal keypad location with a black letter tray. In one of the two test conditions in each test block, the right-hand keypad was relocated to the mold 90° to the side of the participants’ shoulder. The chinrest was placed at 60 cm from the display to diminish any effect of the right hand being relocated over the course of the experiment and to assure that potential head-based frames of reference did not differ across participants. The room (2.25 × 2.25 × 3.50 m) was dimly lit with fluorescent light and fitted with a webcam for monitoring purposes.

### Task

Upon registering for the experiment, participants received the assignment to memorize two six-digit numbers, without telling them why. The ability to recite the numbers from memory was a prerequisite for further participation in the experiment, and one participant was rescheduled to a later time slot for not having learned the numbers. The digits in these six-digit numbers were associated with the left little finger (“1”) through the right little finger (“8”). At the start of the experiment, participants were told that only the first response of a sequence would be indicated by the location of the stimulus on the display, after which the memorized numbers were to be used as imperatives by the participant for the subsequent key presses in the sequence. Presentation of key-specific stimuli was omitted to preclude incompatible mappings between the location of a stimulus and the location of the associated response when the right hand was at the participants’ side.

For the little finger (“pinky”) and the ring, middle, and index fingers of the left hand (“p”, “r”, “m”, and “i”, respectively) and for the index, middle, ring fingers, and the pinky of the right hand (“I”, “M”, “R”, and “P”, respectively), the base sequences were ipIRPm (learned as 4-1-5-7-8-3) and MRmprI (6-7-3-1-2-5). The order of the sequences was counterbalanced over participants across the fingers of each hand. So, ipIRPm was counterbalanced to prMPIi, rmRIMp, and miPMRr, while MRmprI was counterbalanced to RPirmM, PIpmiR, and IMripP. Hence, the transitions between the second and third key presses in each sequence (i.e., R_2_ and R_3_), and between R_5_ and R_6_ involved a between-hand transition for each participant. Given their design, the two sequences are indicated as the LLRRRL and RRLLLR sequences with L indicating a left-hand finger and R a right-hand finger.

Throughout the entire experiment, the display showed eight homogenously gray square outlines (6 × 6 mm) against a black background and with a black filling. This layout corresponds with the spatial arrangement of the assigned response keys (i.e.,/, 8, 5, 2, 3, 6, 9, and *) when the two keypads were in the adjacent frontal location. The eight squares were placed in a horizontal order with 4-mm spacing (i.e., about 0.4° at 60-cm face–display distance). The row of squares was centered in the horizontal plane and vertically aligned at about one third from the top of the display.

The stimulus consisted of one square lighting up by its filling becoming bright green. Participants responded by depressing the spatially compatible key, after which the square became black again. All ensuing key presses were to be deduced from the corresponding, pre-learned six-digit numbers (cf. Experiment 2 in De Kleine and Verwey 2009). The completion of a six-element keying sequence is denoted a trial. Pressing a false key (i.e., failing to execute the sequence in its correct order) terminated a trial and resulted in the message “Wrong key” (in Dutch) being displayed for 500 ms. A premature first response resulted in a message saying “Too early” (in Dutch). The ensuing sequence started 1000 ms after a sequence was completed or terminated. Key release was not registered and could thus follow depressing a later key.

### Procedure

At the start of the experiment, the experimenter helped the participant attain a posture that would remain comfortable even when the right hand was turned 90° outward. An on-screen message instructed participants on which keys they were to place their fingers. At the end of each block, and halfway through each block, participants received feedback for 10 s displaying their average execution time and error rates. If the error rate rose above 8 %, the message “Try to respond more accurately” (in Dutch) was presented. Error rates below 3 % resulted in the message “Try to respond faster” (in Dutch) in order to prevent cautious and therefore slow key pressing.

The experiment consisted of nine blocks in a single session. In Block 1, both keypads were placed in the mold in front of the participant. Each practice block in this experiment was composed of an 80-trial sub-block, a 20-s break, and another 80-trial sub-block. At the end of each block, a message informed the participant the block had finished, that the participant was to wait for the experimenter, and that a 4-min break started. If necessary, the experimenter would encourage the participant to improve sequence execution by responding faster or more accurately.

Block 2 was the first test block. By then, all participants had learned to translate the six-digit code into motor actions. The test block was composed of two 40-trial sub-blocks separated by a break that the experimenter terminated manually. The two sub-blocks of each test block differed in the location of the right keypad, which was either in front of the participant as with the practice blocks, or placed in a mold 90° to the right (relative to the shoulder). Half of the participants started with the right keypad at the side of the body and then performed with the right keypad in front of them, while this was reversed for the other half. Next, the participants continued practice in Blocks 3 through 8 with the two keypads in the frontal position. Finally, Block 9 was the second test block, which was identical to Block 2. Across Blocks 1 through 9, participants performed 600 repetitions of each sequence with the two keypads in the frontal location, and two times 20 trials per sequence with the right-hand keypad relocated to the participants’ side.

Following Block 9, the participants filled out a questionnaire. It first asked participants to write down the two sequences they had been practicing (‘free recall’) in terms of the symbols on the keys they had been depressing. As support, the symbols on the actual keys were presented in the questionnaire in a spatially realistic location (i.e., /852 369*). Notice that these symbols differed from the ones the participants had been learning before the experiment (which had consisted of the numbers 1–8). Next, the participants were asked to indicate the strategy they had been using for writing the sequences down. That is, whether (a) they had remembered the order of the symbols on the keys, (b) they had remembered the order of the stimulus locations, (c) they had executed the sequences with their fingers on the table or in their mind, or (d) they had used some other strategy.

### Data analysis

The first two trials of each sub-block and sequences with an error were excluded from the RT analyses. Sequences were considered outliers and also removed when their total execution time was longer than the average time plus three times the standard deviation in a block. This excluded 1.8 % of the sequences. Errors were computed per block and for each sequential position. Error frequency was arcsin-transformed before being submitted to an ANOVA to stabilize the variance (p. 356 in Winer et al. 1991). The times between successive keying responses are denoted: the sequence initiation interval (T_1_) for the response to the stimulus and interkey intervals T_2_ through T_6_ for the five consecutive responses. Responses are denoted also by indices (R_1_–R_6_).

## Results

### Practice blocks

**Fig. 2:**
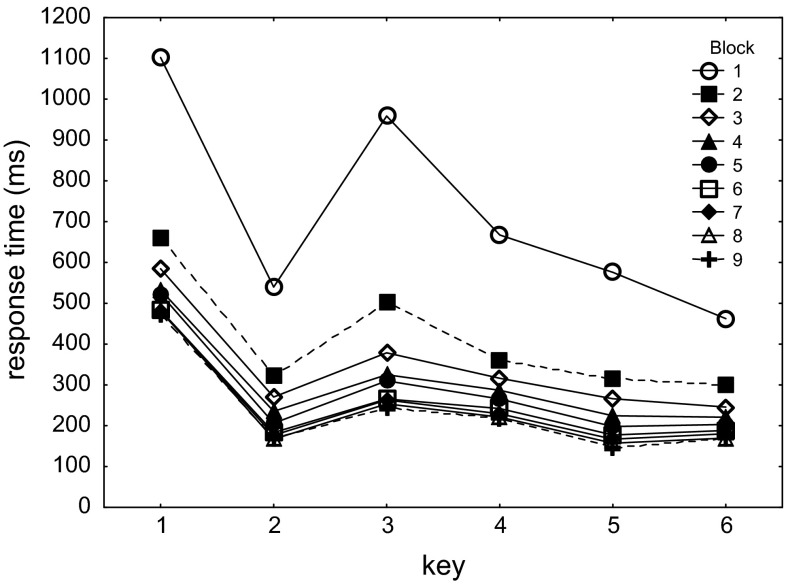
Response times as a function of Block and Key position. Blocks 2 and 9 include only the sub-blocks with the *right-hand* keypad in the frontal location (like in the practice blocks). Between-hand transitions occurred before R_3_ and R_6_

Figure 2 shows the RTs obtained with both hands in the frontal location as a function of Block and Key collapsed across the two sequences. The RTs were analyzed using a 9 (Block) × 2 (Sequence: LLRRRL vs. RRLLLR) × 6 (Key) repeated-measures ANOVA. From Blocks 2 and 9 only the sub-block was included in which participants used the right hand in the normal frontal location. This ANOVA showed main effects of Block, *F*(8,184) = 232.4, *p* < .001, *η*_*p*_^*2*^ = .91, and Key, *F*(5,115) = 67.6, *p* < .001, *η*_*p*_^*2*^ = .75. These effects support the notions that participants improved with practice, and that RTs differed as a function of key position in the sequence. A significant Block × Key interaction confirmed that key presses at some sequential locations gained more from practice than others, *F*(40,920) = 7.8, *p* < .001, *η*_*p*_^*2*^ = .25.

With respect to the effect of the between-hand transitions, Fig. 2 indicates that in Block 1 the second between-hand transition during T_6_ was not slower than the preceding responses. This implies that R_6_ was not treated as a separate “1-element chunk” and, hence, that a between-hand transition does not necessarily lead to a clear segmentation of a movement sequence. In Block 1, T_6_ was even shorter than T_5_, *F*(1,23) = 6.9, *p* = .02, *η*_*p*_^*2*^ = .23, but this advantage of T_6_ reduced with block, *F*(1,23) = 7.1, *p* < .001, *η*_*p*_^*2*^ = .24, and it was no longer significant across Blocks 2–9, *F*(1,23) = 0.9, *p* = .34, *η*_*p*_^*2*^ = .04.

Figure 2 shows also that the first between-hand transition during T_3_ was indeed relatively slow in the earlier blocks. Planned comparisons indicated that while R_3_ was significantly slower than R_2_ and R_4_ together in each of the 9 blocks, Fs(1,23) > 5.7, *p*s < .03, *η*_*p*_^*2*^s > .20, in later blocks this effect can be attributed to the fast R_2_ and not to R_4_. T_3_ was significantly longer than T_4_ only in Blocks 1 and 2, Fs(1,23) > 6.6, *p*s < .02, *η*_*p*_^*2*^s > .22, and this T_3_–T_4_ difference was indeed significantly greater in Blocks 1–4 than in Blocks 6–9, *F*(1,23) = 7.5, *p* = .01, *η*_*p*_^*2*^ = .25. Given that the fast R_2_ can be attributed to response preparation on the basis of explicit sequenced knowledge (Verwey 2015), which was high (see below), these results support initial segmentation at T_3_, but not at T_6_, and further show that the initially slow R_3_ did not last with practice. Only the fast R_2_, relative to R_3_, persisted until Block 9, *F*(1,23) = 10.4, *p* = -.003, *η*_*p*_^*2*^ = .31.

Arcsin-transformed error proportions per key were subjected to the same repeated-measures ANOVA as used for the RTs. Only the first error in a sequence counted because execution of the sequence was terminated after an error. The ANOVA showed main effects of Block, *F*(8,184) = 17.1, *p* < .001, *η*_*p*_^*2*^ = .43, and Key, *F*(5,115) = 6.4, *p* < .001, *η*_*p*_^*2*^ = .22, and a Sequence × Block interaction, *F*(8,184) = 2.7, *p* = .008, *η*_*p*_^*2*^ = .11. These results indicated a relatively high error rate in Block 1 (of 3.3 % per key), and in Block 8 (2.2 % per key), while the other error proportions per key were below 2 %. The error rate was high for R_3_ relative to the other keys (1.9, 1.1, 2.6, 2.2, 2.2, and 1.3 %, respectively). Finally, according to the above-mentioned Sequence × Block interaction, LLRRRL had a relatively high error rate in Block 2 compared to RRLLLR (1.7 vs. 1.0 % per key), whereas error rates tended to be somewhat higher for RRLLLR in Blocks 4, 5, and 7.

### Test blocks: representing movement sequences

**Fig. 3:**
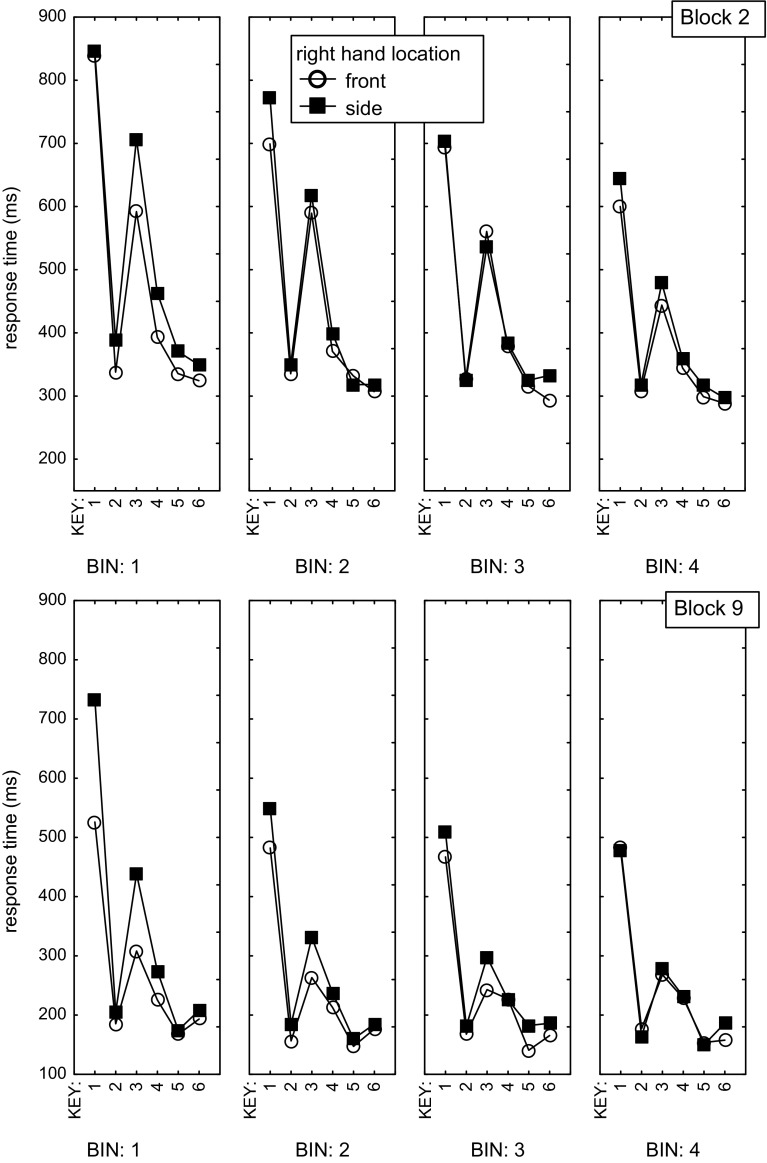
Individual RTs in Bins 1–4 of Blocks 2 and 9 pooled across the two *right-hand* location conditions

To analyze the rate at which RT changed within the test blocks after the between-hand transitions, we analyzed performance in Block 2 (i.e., after 80 or 100 practice trials per sequence), and in Block 9 (after 580 or 600 practice trials) by distinguishing four successive five-trial bins. The averages of the errorless sequences in these bins were therefore analyzed with a 4 (Bin) × 2 (Block: 2 vs. 9) × 2 (Sequence: LLRRRL vs. RRLLLR) × 2 (Location of the right hand: Front, Side) × 6 (Key) repeated-measures ANOVA (see Fig. 3). In addition to the typical main effects of Block, *F*(1,23) = 134.6, *p* < .001, *η*_*p*_^*2*^ = 0.85, and Key, *F*(5,115) = 52.2, *p* < .001, *η*_*p*_^*2*^ = .69, this ANOVA showed main effects of Bin, *F*(3,69) = 41.6, *p* < .001, *η*_*p*_^*2*^ = .64, and Location, *F*(1,23) = 10.5, *p* = .004, *η*_*p*_^*2*^ = .31. The sequence main effect was not significant, *F*(1,23) = 1.6, *p* = .21, *η*_*p*_^*2*^ = .07, and neither were all other interactions that included sequence and location, with the exception of the highest-order Bin × Block × Sequence × Location × Key interaction, *F*(15,345) = 2.0, *p* = .01, *η*_*p*_^*2*^ = .08). These results suggest that location had similar effects on the LLRRRL and RRLLLR sequences.

According to the significant Key × Location interaction, relocating the right hand slowed some sequence elements more than others, *F*(5,115) = 2.6, *p* = .03, *η*_*p*_^*2*^ = .10 (the location effect amounted to 55, 15, 52, 24, 13, and 20 ms for R_1_–R_6_, respectively). This is inconsistent with the applicability hypothesis and supports the adjustment hypothesis. A planned comparison confirmed that this interaction was a result of a larger location effect on responses that according to the adjustment hypothesis are preceded by an adjustment of the spatial sequence representation (i.e., R_136_), than on the other responses (R_245_), *F*(1,23) = 7.1, *p* = .01, *η*_*p*_^*2*^ = .24. Further support for the adjustment hypothesis comes from the significant Bin × Location interaction which shows that the slowing that resulted from relocating the right hand was reduced across successive bins (61, 29, 17, 12 ms, respectively), *F*(3,69) = 3.0, *p* = .04, *η*_*p*_^*2*^ = .12. This rapid reduction in the location effect is incongruent with the applicability hypothesis, as that would require a new representation which would probably take many more than the 20 trials per sequence available in each test block to develop. The reduction in the location effect for R_136_ across successive bins was not supported by a Key × Bin × Location interaction in the omnibus ANOVA, *F*(15,345) = 0.99, *p* = .47, *η*_*p*_^*2*^ = .04 [but it was significant in a Block 9 ANOVA, *F*(3,69) = 2.8, *p* = .05, *η*_*p*_^*2*^ = .11].

Figure 3 shows that the location effect had been caused by R_1_ and R_3_, especially in the earlier bins of Block 9, and not by R_6_. This was not unexpected given that the last response of a six-key sequence (just like R_2_) has been found to disproportionally benefit from explicit sequence knowledge (Verwey 2015). This notion was supported by a planned comparison across both test blocks and sequences showing that the location effect was greater for R_13_ than for R_6_, *F*(1,23) = 5.6, *p* = .03, *η*_*p*_^*2*^ = .20.

Given the above support for the adjustment hypothesis, we then used planned comparisons to examine the prediction of that hypothesis that adjustment should be stronger in Block 9 than in Block 2 (because execution in Block 9 involves more task-specific representations). In line with this prediction, the location effect in R_13_, relative to R_245_, was significant across all bins of Block 9, *F*(1,23) = 11.9, *p* = .002, *η*_*p*_^*2*^ = .34, while this was not the case with Block 2, *F*(1,23) = 0.6, *p* = .63, *η*_*p*_^*2*^ = .03. Furthermore, the location effect in R_13_ (relative to R_245_) was significantly larger in Block 9/Bin 1 than in Block 2/Bin 1, *F*(1,23) = 4.7, *p* = .04, *η*_*p*_^*2*^ = .17. This Block difference did not reach significance across all fours bins, *F*(1,23) = 2.4, *p* = .13, *η*_*p*_^*2*^ = .09, which is reasonable given that the adjustment hypothesis suggests that the location effect reduces quite quickly within a test block. Indeed, a Location × Bin interaction in the Block 9 ANOVA reflected a reducing location effect across successive bins, *F*(3,69) = 4.0, *p* = .01, *η*_*p*_^*2*^ = .15, though the location effect was still significant across Bins 2–4, *F*(1,23) = 5.3, *p* = .03, *η*_*p*_^*2*^ = .19.

While the location effect was significant for R_13_ across both sequences, according to the adjustment hypothesis the location effect should be larger in R_1_ of RRLLLR than of LLRRRL, especially in Block 9/Bin 1 when representations are more task-specific than in Block 2. This prediction is supported by the earlier mentioned Bin × Block × Sequence × Location × Key interaction in the omnibus ANOVA, *F*(15,345) = 2.0, *p* = .01, *η*_*p*_^*2*^ = .08. A planned comparison to test this specific hypothesis showed that in Bin 1 of Block 9, R_1_ of RRLLLR was substantially slower when carried out by the relocated right hand than R_1_ of LLRRRL (856 vs. 581 ms, respectively), *F*(1,23) = 4.6, *p* = .04, *η*_*p*_^*2*^ = .17. Also, in Block 9/Bin 1 R_1_ was substantially slower with the right hand at the side than in the front, *F*(1,23) = 5.1, *p* = .03, *η*_*p*_^*2*^ = .18. Most likely because of the limited statistical power of higher-order interactions, this effect was not significantly greater in Block 9/Bin 1 than in Block 2/Bin 1, *F*(1,23) = 2.8, *p* = .11, *η*_*p*_^*2*^ = 11.

Arcsin-transformed error proportions in Blocks 2 and 9 were analyzed using a 2 (Block: 2, 9) × 2 (Location) × 6 (Key) repeated-measures ANOVA. It showed main effects of Block, *F*(1,23) = 6.4, *p* = .02, *η*_*p*_^*2*^ = .22, and Key, *F*(5,115) = 3.8, *p* = .003, *η*_*p*_^*2*^ = .14, indicating a slight increase in errors after practice (1.4 vs. 2.0 % per key), and some variation across key positions (1.6, 0.7, 2.4, 2.0, 2.1, and 1.5 %, respectively). The LLRRRL sequence had a higher error rate than RRLLLR with the right hand in the frontal location (1.8 vs. 1.3 %), and a lower error rate with the right hand on the side (1.7 vs. 2.2 %), *F*(1,23) = 14.1, *p* = .001, *η*_*p*_^*2*^ = .38. The Location × Key interaction, *F*(5,115) = 3.3, *p* = .009, *η*_*p*_^*2*^ = .13, indicated that with the right hand on the side, error proportion was a little higher for R_1_ (frontal: 1.3 % vs. side: 1.9 %), and R_4_–R_6_ (1.4 vs. 2.3 %), whereas it was lower for R_3_ (2.8 vs. 1.9 %).

Taken together, these analyses showed several findings in support of the adjustment hypothesis: Two of the responses that according to the adjustment hypothesis were preceded by adjustment (R_13_) were slowed more by the right-hand relocation than the other responses (R_245_). This slowing was greatest after more extensive practice in Bin 1 of Block 9 after which it reduced in the ensuing five-trial bins. However, R_6_ did not show any effects of relocating the right hand. This can be attributed to the use of explicit sequence knowledge. Finally, the location effect on R_1_ in the RRLLLR sequence appeared quite large, especially in Bin 1 of Block 9 (the expectation that this R_1_ would be relatively slow was not supported by all associated higher-order interactions which is probably due to a lack of statistical power).

### Test blocks: chunking boundaries in Blocks 2 and 9

We also examined the effect of the between-hand transitions in Blocks 2 and 9 in the condition in which the right hand was in the frontal location. We used the R_5_–R_6_ difference and the R_3_–R_4_ difference to explore whether the first response following a between-hand transition might have induced the start of a segment that eventually consolidated (we excluded R_2_ from the last comparison because that response was most likely sped up by explicit knowledge, Verwey 2015). It immediately stood out that R_6_ was never slower than R_5_, not even in Block 1 (see Fig. 1). In contrast, the first between-hand transition appeared to be followed by a slow R_3_, that in Block 2 was 171 ms slower than R_4_, *F*(1,23) = 7.4, *p* = .01, *η*_*p*_^*2*^ = .24. Detailed examination of the individual participants’ RTs per sequence showed that in Block 2 R_3_ was slower than R_4_ in only 30 of the 48 sequences (each of the 24 participants carried out 2 sequences). So, the between-hand transition certainly had no consistent effect on all participants. In Block 9, the R_3_–R_4_ difference (of 44 ms) did not reach significance any more, *F*(1,23) = 2.3, *p* = .14, *η*_*p*_^*2*^ = .09, and R_3_ appeared slower than R_4_ in only 25 of the 48 sequences. So, like the second between-hand transition, the first between-hand transition did not seem to induce a concatenation point.

### Awareness

We examined the numbers of participants who correctly recorded the six elements of 0, 1, or 2 sequences. Table 1 shows that 22 of the present 24 participants (92 %) had full awareness of two six-key sequences that were practiced. This was unexpectedly high given that the participants in five recent studies—which involved display of key-specific stimuli—showed that significantly less participants (namely 47 %) had been fully aware, χ^2^(2) = 103.3, *p* < .001 (these studies were reported in Ruitenberg et al. 2012; Verwey 2015; Verwey and Abrahamse 2012; Verwey et al. 2010; Verwey and Wright 2014). Due to this high awareness, we could not assess whether performance correlated with awareness (especially of R_2_ and R_6_).

**Table 1:** Numbers (and percentages) of participants correctly recalling (by writing freely) 0, 1, or 2 of their two six-key sequences in the present and in the five earlier DSP studies (see text), and the numbers (and percentages) of participants who indicated to have used a particular strategy to write down their two six-key sequences in the present and in four or five earlier DSP studies (see text for details)

	Present study (*n* = 24)	Five recent studies (*n* = 144)
Free recall: sequences correct		
0	0 (0 %)	38 (26 %)
1	2 (8 %)	39 (27 %)
2	22 (92 %)	67 (47 %)

	Present study (*n* = 24)	4 recent studies (*n* = 93)
Awareness strategy		
Knew spatial positions	4 (17 %)	21 (23 %)
Used finger tapping	12 (50 %)	63 (68 %)
Knew letters/symbols	8 (33 %)	9 (10 %)

Half of the present 24 participants (50 %) indicated that they had reconstructed their explicit knowledge when filling out the questionnaire by executing the sequences on the table top or covertly in their heads (Table 1). This number of reconstructing participants was significantly less than across four previous studies (Verwey 2015; Verwey and Abrahamse 2012; Verwey et al. 2010; Verwey and Wright 2014) where 68 % of the participants indicated to have used this strategy, χ^2^(2) = 55.2, *p* < .001. On the one hand, these numbers indicate that, despite the capacity of most participants to write down their sequences, still half of them had no direct access to their sequence knowledge. On the other hand, given that explicit knowledge of discrete keying sequences is highest at the start and end of a sequence due to primacy and recency effects (Verwey 2015), the high awareness in the present study show that it is quite likely that the present participants could speed up R_6_ using explicit knowledge.

## Discussion

In order to better understand the nature of the representations that make up the motor chunks used to execute DSP keying sequences, we explored in detail how execution of two bimanual, familiar keying sequences would be affected when the right hand is relocated to the side. Explicit sequence knowledge was assessed to see whether that kind of sequence knowledge might be used to counteract a potential hand relocation effect on, especially, R_6_ (Verwey 2015). We further explored whether the between-hand transitions between R_2_ and R_3_ and between R_5_ and R_6_ would induce segmentation of the sequence in early practice, and whether these would eventually consolidate into concatenation points of successive motor chunks.

### Adjusting spatial representations

The present data support an adjustment hypothesis that assumes that practice induces highly task-specific representations that can be adjusted when one of the hands is in a new location. They are inconsistent with the applicability hypothesis that asserts that inapplicable representations are no longer used.^2^

The support for the adjustment hypothesis comes from the findings that relocating the right hand affected mostly the first response carried out by each hand (i.e., R_1_ and R_3_ in RRLLLR, and R_3_ in LLRRRL—but not R_6_, see below), and that this slowing lasted only about 15 trials. This slowing suggests that before a hand could start executing its part of the keying sequence, the sequence representation had to be adjusted to the location of that hand. The finding that this effect reduced so quickly in the successive bins of the test blocks shows that participants quickly were able to perform this adjustment more efficiently. This efficiency may be based on performing the adjustment during execution of the preceding responses (Verwey 1995, 2001), but the observation that even in RRLLLR R_1_ rapidly became faster (in which case preparation could not be carried out during earlier responses) suggests that the adjustment itself was quickly learned. Such a rapid adjustment is in line with neurophysiological studies showing adjustment of neural representations of space in less than 50–100 ms (Derdikman and Moser 2010; Georgopoulos et al. 1986; Zacks 2008).

The adjustment hypothesis is not supported by the observation that R_6_ did not show a hand location effect. It is possible that adjustment of R_6_ was easy because it involved just a single response and could entirely occur during execution of the preceding responses. Indeed, preparing one response during a preceding keying sequence seems to proceed more smoothly than when a few responses are prepared during the preceding key presses (Verwey 1995, 2001). Alternatively, the high awareness in the present participants of their sequences and the high saliency of the last response (one key press that was executed with another hand) may imply that the participants used explicit knowledge to speed up the last response rather than that they adjusted the sequence representation for just a single last response. This possibility is in line with the recent finding that especially the last response of a six-key DSP sequence is sped up by using explicit sequence knowledge (Verwey 2015). Preparing explicit knowledge of R_6_ could probably occur during execution of earlier responses because after practice, the central processor was no longer required for executing the sequence (Abrahamse et al. 2013; Verwey et al. 2015).

The current pattern of results provides some indications as to the nature of the motor chunks. The observation that sequence execution by both hands suffered equally from relocating one hand suggests that after about 600 trials the sequence representation relied primarily on a cross-hand trunk- or head-based reference frame that was adjusted at each between-hand transition. While neurophysiological research provided evidence for the existence of hand-based reference frames (Zacks 2008), the present finding that relocating one hand slowed both hands suggests that sequence execution did not rely much on such hand-specific, egocentric representations. Basically, allocentric representations may have played a role as well, but these are not believed to play an important role at advanced levels of movement execution (Willingham 1998). Earlier findings that execution rate is slowed when a sequence is carried out by other effectors, should probably be attributed to a spatial representation that is also effector-specific (Andresen and Marsolek 2012; Park and Shea 2005; Verwey and Clegg 2005; Verwey and Wright 2004), rather than to a muscle-oriented representation (Hikosaka et al. 1999; Keele 1968; Shea et al. 2011; Verwey et al. 2015). This dominant role of an effector-specific spatial representation is in line with findings with DSP sequences that execution rate reduced more when fingers of the other hand were used (Verwey and Wright 2004) than adjacent fingers of the same hand (Verwey et al. 2009). Participants in the latter study using adjacent fingers seem to have been able to make better use of the spatial hand-specific representations than in the former study where the other hand was being used. It should be noted, however, that the present results do not necessarily reject the possibility that sequence coding did include a component that was muscle specific or in a hand-based reference frame. For example, early in Block 9, participants may have relied on such a hand-based representation. Still, the dominant representations in Block 9 seem to have been one coded in a cross-hand trunk- or head-based spatial reference frame.

The finding that R_1_ and R_3_ in RRLLLR were both slowed indicates that a sequence representation was activated in memory, and it was then adjusted before executing R_1_ and then adjusted again before executing R_3_. In terms of motor buffer models, like the dual processor model (Verwey 2001), this shows that motor chunk representations that have already been loaded into the motor buffer can still be adjusted to different spatial conditions. Apparently, once loaded the motor buffer remains open to cognitive manipulations.

### No consolidation of between-hand transitions

We further examined whether segmentation with limited practice may be determined by salient between-hand transitions and whether this would consolidate into motor chunks with practice. It was immediately obvious that R_6_ was never faster than R_5_. And while across all participants R_3_ appeared to be significantly slower than R_4_ in Block 2, this pattern occurred in only about two-thirds of the 48 averaged sequences per participant and block (2 sequences per participant in each block). In Block 9 the R_3_–R_4_ difference did not reach statistical significance at all. These results reject the hypothesis that in discrete keying sequences a between-hand transition is an important determinant of segmentation and chunking. This endorses the idea that motor chunks—at this level of practice—do not include hand-based representations, but include cross-hand representations.

## Conclusions

The present study indicates that after 600 practice trials per sequence, motor chunks in a DSP task rely heavily on a cross-hand representation that codes individual key presses in a trunk- or head-based spatial reference frame. Recent indications that explicit knowledge is especially important for the last response in a six-key sequence (Verwey 2015) suggests that the lack of a hand location effect on R_6_ was caused by the use of explicit knowledge. After these 600 practice trials, it took only about 15 trials to fully adjust this representation to a novel location of the right hand. This spatial sequence representation seems to be implicit in the sense that, despite the high scores on an awareness test, half of the participants indicated that to write down the sequence elements they had to reconstruct their sequences by mentally or physically “executing” them. No indication was found for a clear contribution of a muscle-based (motor) representation or a hand-based spatial representation as that should have made execution rate of all sequence elements insensitive to the changed right-hand location. Indications for effector-specific learning—previously taken as evidence for motor learning—can perhaps be explained better by an adjustment of the timing to the biomechanical properties of the effectors used (Park and Shea 2003; Shea and Kovacs 2013). Nevertheless, a role of hand-based representations cannot be excluded yet. Such a representation may have been responsible for executing the sequences in the trials immediately after the hand had been relocated. Finally, the present data indicate that a between-hand transition may perhaps influence how some participants strategically segment their sequences in early practice, but there are no indications that these transitions determine the sequential positions at which successive motor chunks are eventually concatenated.

## Footnotes

1 Segmentation involves the perceivable grouping of elements that probably reflects some cognitive strategy, whereas motor chunking explicitly suggests the use of integrated sequence representations (Verwey and Eikelboom 2003).

2 Because the adjustment hypothesis predicts effects especially after substantial practice, we performed also an ANOVA on just Block 2 results which is not reported here. In that block, the location effect did not reach significance, neither across all responses nor for some responses alone. This is consistent with the notion that performance in Block 2 was still largely based on sequence representations that are not hand location-specific (like the learned 6-digit numbers). In other words, the lack of significant location effects in Block 2 supports the assumption of the applicability hypothesis that the location effect in Block 9 is based on slower developing representations.
